# Left ventricular myocardial functions in pediatric patients with primary nephrotic syndrome: a comprehensive evaluation using conventional echocardiography, two-dimensional speckle tracking, and three-dimensional echocardiography

**DOI:** 10.1186/s13052-025-02086-5

**Published:** 2025-07-15

**Authors:** Amira Hussein, Shaimaa Rakha, Ayman Hammad, Mona Hafez, Mai S Korkor

**Affiliations:** 1 Pediatric cardiology Unit, Faculty of Medicine, Mansoura University, Mansoura, Egypt; 2 Pediatric nephrology Unit, Faculty of medicine, Mansoura University, Mansoura, Egypt

**Keywords:** Nephrotic syndrome, Left ventricle, 3D echocardiography

## Abstract

**Background:**

Nephrotic syndrome (NS) has been linked to cardiac morbidity and mortality. However, early cardiac implications in NS were studied on a limited scale using basic echocardiography (ECHO). Therefore, the study aimed to evaluate subclinical Left ventricle (LV) functional alterations using conventional and advanced ECHO modalities.

**Methods:**

A prospective observational study was conducted from January 2022 to August 2024 on 40 primary NS patients and 40 controls. Demographic, clinical, and laboratory data were collected. ECHO was performed, including LV functional assessment using conventional ECHO, tissue Doppler, two-dimensional (2D) speckle tracking, and three-dimensional (3D) ECHO.

**Results:**

NS patients, whose mean age was 10.93 ± 2.78 years, were subclassified into steroid-sensitive NS (SSNS) 15(37.5%) and steroid-resistant NS (SRNS) 25 (62.5%). Compared with controls, there was no significant difference regarding conventional ejection fraction (EF) or E/A ratio. However, E/E’ ratio and tissue Doppler Tie index were significantly higher in NS (* p* = 0.001, *p*= < 0.001, respectively), particularly SRNS. Average global longitudinal strain (GLS) was significantly lower in NS (*p* = < 0.001), especially SRNS, while 3D ECHO-measured EF significantly declined in NS (*p* = < 0.001). Tei index and E/E’ were moderately correlated with current cholesterol level, while E/E’ and GLS correlated with initial serum albumin. On regression analysis, current cholesterol and initial serum albumin were significant predictors for E/E’;  *p*= 0.019 (ß: 0.014, 95% CI: 0.003–0.26) and  *p*= 0.003 (ß:-1.41, 95% CI: -2.28- -0.53), respectively. No significant factors predicted GLS or 3D EF.

**Conclusion:**

In addition to diastolic subclinical LV dysfunction in children with primary NS detected using Tissue Doppler, systolic alterations could be detected using 2D speckle tracking or 3D ECHO. Some of the resultant subtle LV-impaired parameters could be correlated to NS-related biochemical changes.

## Introduction

Nephrotic syndrome (NS) is the leading cause of chronic kidney disease in children, with its known characteristic features of heavy proteinuria, hypoalbuminemia, and/or edema [1, 2] Steroids are the main line of treatment for primary NS, with complete remission of proteinuria occurring in 80–90% of patients who are labeled as steroid-sensitive NS (SSNS) [3]. The remaining portion of patients who do not achieve full remission are labeled as steroid-resistant NS (SRNS) and require other immunosuppressive drugs, such as calcineurin inhibitors (CNI) [4].

Patients with NS have an increased risk of cardiovascular events such as heart failure and acute coronary syndrome, end-stage renal disease, and death [5]. In addition to the disease itself, the use of steroids and CNI has many adverse effects [6]. Steroids lead to several complications that impact heart functions profoundly, such as obesity, hypertension, and impaired glucose tolerance [7, 8]. Moreover, cardiac remodeling due to oxidative stress, endothelial dysfunction, and steroid toxicity could occur and lead to cardiac dysfunction and thromboembolism [7, 8].

Routine evaluation of left ventricle (LV) myocardial functions using conventional echocardiographic modalities was found to be limited in detecting subclinical impairments. However, two-dimensional speckle tracking has been found to be effective in diagnosing early deterioration in LV functions despite apparently normal traditional echocardiography (ECHO) parameters [9, 10]. Moreover, with the development of three-dimensional echocardiography (3DE), complete comprehensive LV volumetric and functional assessment are feasible with automated generation of results that are comparable to cardiac magnetic resonance. On the other hand, conventional methods could be limited by potential foreshortening and out-of-plane wall motion abnormalities [11, 12].

Nevertheless, Limited research studied the changes in LV myocardial function in NS using advanced echocardiographic modalities, especially two-dimensional speckle tracking and 3DE [10, 13]. Therefore, the aim of this study was to evaluate the subclinical LV functional alterations in patients with primary NS compared to age-matched healthy controls.

## Methods

### Study design

The study is a prospective case-control study that included 80 participants. Children with primary NS, either SSNS or SRNS, were recruited between January 2022 and August 2024 from the nephrology outpatient clinic at Mansoura University Children’s Hospital, Egypt, a tertiary referral center with all pediatric subspecialties. Written informed consent for enrolment in the study was obtained from all participants’ legal guardians. The study protocol was approved by Mansoura University Faculty of Medicine Institutional Research Board (IRB).

### Inclusion criteria

Primary NS patients aged 1–18 years who were diagnosed at least one year before recruitment were included. The patients were diagnosed based on the criteria of the International Study for Kidney Disease in Childhood (ISKDC) for the diagnosis of NS [14]. All included subjects were in remission, defined as three consecutive days of trace or negative protein in urinalysis. The recruited patients had normal serum complement C3 and normal kidney function, defined as an estimated glomerular filtration rate above 90 ml/min /1.73m2 (calculated by Schwartz formula) [15]. Age-matched healthy controls were included for comparison purposes.

### Exclusion criteria

Patients with known hemodynamically significant congenital heart diseases, coronary artery disease, primary cardiomyopathies, and arrhythmias were excluded. Moreover, cases of impaired kidney function or those diagnosed with a systemic disease known to impair cardiac functions were not included. Another exclusion criterion was inadequate echocardiographic image quality that interfered with the 3D processing. Patients with secondary causes of NS, such as systemic lupus and diabetes mellitus, were excluded.

### Sample size calculation

The study’s sample size was calculated using G*power version 3.1.9.7. The configured sample consisted of 32 participants for the patients’ group and for the control group, at a 5% level of significance and 85% power of the study. The calculation was based on using the mean and SD of LV GLS results of cases and controls by Çap et al. [10]. The sample was increased to 40 in each group to adjust for incomplete data and increase study power.

### Demographics, disease course and therapy

All the included patients were subjected to a detailed history, including age, gender, duration of NS, response to steroid therapy, frequency of relapses per year, and treatment protocol at the time of recruitment.

All patients were maintained on steroid therapy (prednisolone), and all SRNS were maintained on ciclosporin therapy with dose adjusted according to the drug trough level and proteinuria assessment. Additionally, some SRNS patients were maintained only on mycophenolate mofetil (MMF) or in combination with ciclosporin. Moreover, most of the patients were maintained on statins to regulate dyslipidemia and angiotensin-converting enzyme inhibitors (ACEI) as anti-proteinuric.

### Anthropometrics and vitals

The body weight, height, and Body mass index (BMI) were measured. BMI was calculated using the standard formula weight (kg)/height2(m2). Blood pressure (systolic and diastolic) in mmHg and heart rate (beat/minute) were evaluated before ECHO performance.

### Laboratory data

The serum levels of creatinine, albumin, cholesterol, complement C3, and urinary albumin/creatinine ratio (ACR) at the time of initial diagnosis and at the time of current cardiac evaluation were collected from patients’ electronic records.

### Echocardiography

All the children’s transthoracic echocardiography (TTE) recordings were performed using a standard ECHO machine, EPIQ CVx Release 5.0 (Philips Medical Systems, Bothell, WA, USA 2018), equipped with an X5-1 matrix array transducer (5 − 1 MHz).

**Fig. 1:**
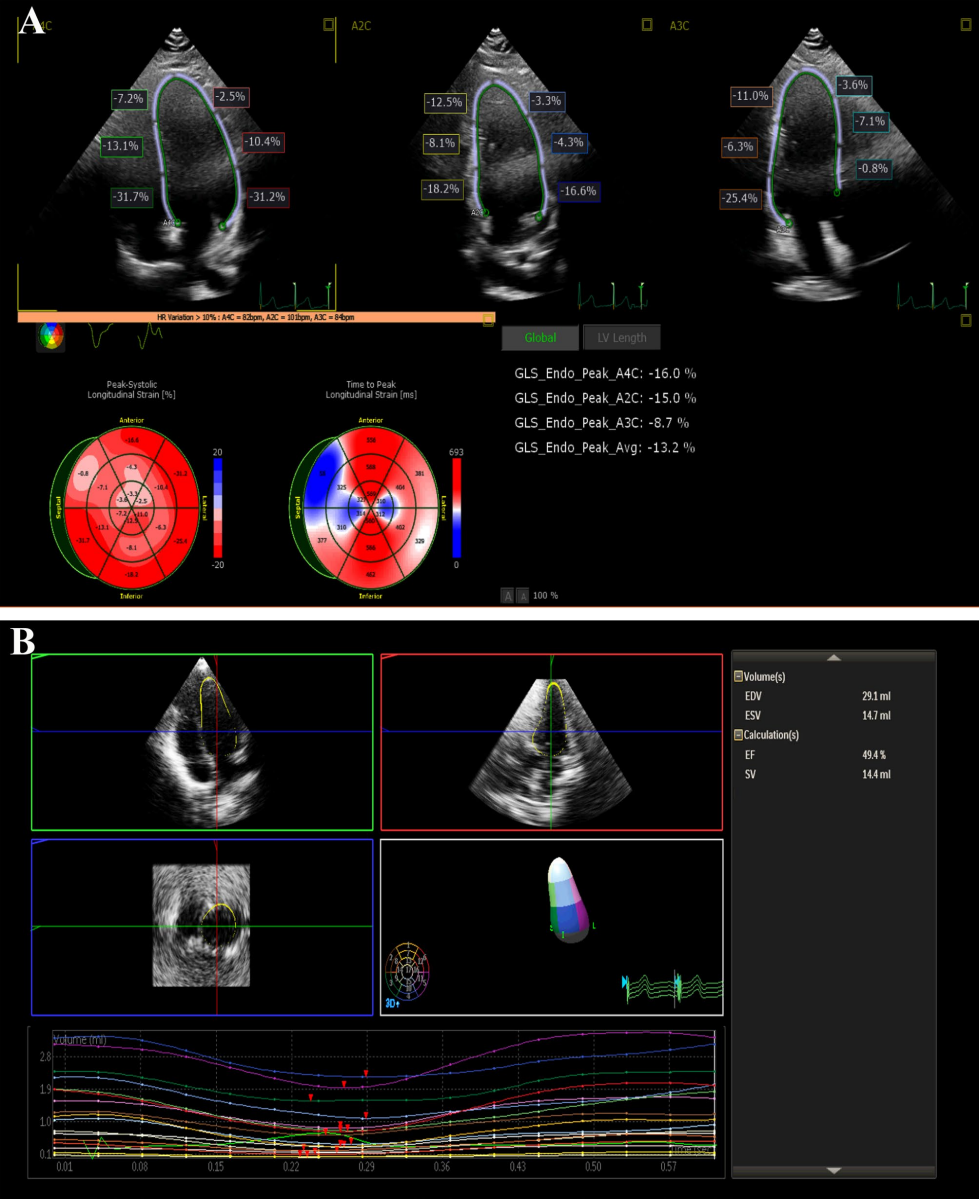
Advanced LV functional assessment in patients with SRNS. Panel **A**: 2D speckle tracking of four-, three-, two-chamber views with auto calculation of GLS and Bull’s eye demonstration; significant low GLS average of -13.2%. Panel **B**: 3DE demonstrating 3D-derived EF of 49.4% auto-calculated.

1. **Conventional ECHO**: Two-dimensional TTE with color Doppler and M.mode evaluation was performed according to the recommendations of the American Society of Echocardiography [16]. M-mode was obtained from two-dimensional parasternal views for internal LV diameter, Interventricular septum, and LV posterior wall thicknesses in millimetres in both systole and diastole, with documentation of LV mass index. LV systolic functions were calculated using fraction shortening (FS) and ejection fraction (EF).
2. **Conventional pulsed Doppler**: Pulsed-wave Doppler was used to assess mitral valve early diastolic flow (E-wave) and late diastolic flow (A-wave) velocities, with E: A ratio estimation.
3. **Tissue Doppler**: Early diastolic (E’ wave), late diastolic (A’ wave), and systolic (S’ wave) velocities were measured at the lateral parts of the mitral annulus on the apical four-chamber views by pulsed-wave tissue Doppler (all in centimeter per second). The E/E’ ratio was calculated. Tei index of LV lateral wall derived from tissue Doppler was determined using the following formula: isovolumic contraction time (IVCT) + isovolumic relaxation time (IVRT)/ET, where ejection time (ET) represents the ejection time of LV from the beginning to the end of S’ wave. Three measurements were averaged for each subject.
4. **Two-dimensional (2D) speckle tracking**: Three LV-focused views (apical four, apical three, and apical two-chamber) were acquired and then selected for analysis using Auto Strain LV software. The Auto Strain LV automatically detects and tracks the contours, and then, after advancing to the analysis step, global longitudinal strain (GLS) and longitudinal strain per apical view are demonstrated.
5. **Three-dimensional volumetric and functional assessment of LV**: Heart Model Acquisition mode (HM ACQ) was used to acquire a full volume 3D dataset at LV-focused four chambers view. All 3D volumes were obtained in a digital format and stored for analysis by dedicated automated quantification software ‘Dynamic HeartModel A.I’ for LV. The software allows LV volumetric and functional quantification. The software tracks every frame over the cardiac cycle using 3D speckle technology to provide a holistic view of the left ventricle function. With automated border detection, the software automatically generates the following 3DE-derived parameters: end-diastolic volume (EDV), indexed End diastolic volume (EDVi), End systolic volume (ESV), EF, and Stroke Volume (SV). An example of 3DE of a SRNS case is demonstrated in Fig. 1.

### Statistical analysis

The collected data were analyzed using the Statistical Package for Social Science (IBM SPSS Statistics for Windows, Version 25.0. Armonk, NY: IBM Corp.). Chi-Square test was used to compare two qualitative variables. Fisher Exact or Monte Carlo test was used for two qualitative variables when the expected count was less than 5 in more than 20% of cells. The statistical significance of the difference between the two study groups was assessed by the Student T-test and Mann-Whitney Test in parametric and non-parametric variables, respectively. The Kruskal-Wallis Test was used to assess the statistical significance of the difference of a non-parametric variable between more than two study groups. The *p*-value was considered significant when < 0.05. Linear correlations were assessed using Spearman correlation coefficients (rho). If rho was from 0 to ± 0.20, it was considered negligible; from ± 0.21 to ± 0.40 weak, from ± 0.41 to ± 0.60 moderate, from ± 0.61 to ± 0.80 strong, and from ± 0.81 to ± 1.00 was considered very strong [17].

## Results

Between January 2022 and August 2024, 40 pediatric patients with NS and 40 healthy controls were enrolled in the study. The NS patients, whose mean age was 10.93 ± 2.78 years, were further subclassified into SSNS 15 (37.5%) and SRNS 25 (62.5%).

The demographic characteristics and clinical data of the study groups are summarized in Table 1. Patients and controls had matching age distribution. Regarding gender distribution, the NS group was significantly associated with a higher male proportion when compared to the control group (*p* = 0.022), with a significant difference noted with a higher percentage of males in the SSNS group compared to the control and SRNS groups. The weight and BMI were significantly increased in NS cases compared to healthy subjects, especially SRNS, in comparison with the control and SSNS groups.

**Table 1:** Demographic and anthropometric data of the studied groups

	Control (*n* = 40)	SSNS (*n* = 15)	SRNS (*n* = 25)	p1	p2	Pairwise		
						p3	p4	p5
Age (years)	10 (7–15)	11 (8–12.5)	12 (4–15.5)	0.25	0.61	0.52	0.24	0.11
Gender (Male)	19 (47.5%)	14 (93.3%)	15 (60%)	0.008*	0.022*	0.002*	0.33	0.03*
Weight (kg)	31 (25–56)	40 (25–50)	57 (16–90)	0.005*	0.039*	0.88	0.003*	0.013*
Height (cm)	134 (125–155)	153 (126–155)	152 (96–172)	0.31	0.37	0.67	0.12	0.38
BMI (kg/m^2^)	16.6 (14.9–23.3)	17.1 (15.8–20.8)	23.8 (16–31.6)	< 0.001*	0.001*	0.81	< 0.001*	< 0.001*
Surface area	1.08 (0.93–1.58)	1.3 (0.94–1.47)	1.57 (0.65–2.09)	0.02*	0.12	0.67	0.013*	0.02*

**BMI**: body mass index, **SSNS**: Steroid sensitive nephrotic syndrome, **SRNS**: steroid resistant nephrotic syndrome

Data in columns of control, SSNS and SRNS are presented as median (IQR) or number (Percentage)

**p1**: Comparing the three studied groups, **p2**: Comparing Control and NS, **p3**: Comparing control and sensitive, **p4**: Comparing control and resistant, **p5**: Comparing sensitive and resistant

*: Significant *p*- value

Table 2 provides a detailed comparison between SSNS and SRNS groups regarding clinical, therapeutic, vital signs, laboratory, and pathologic data. Although the median number of relapses per year was non-significant between groups, hospitalizations were higher in the SRNS than in the SSNS group. Hypertension prevalence significantly differed as all detected cases are in the SRNS category. Regarding laboratory investigations, the initial serum albumin level was significantly lower in SRNS, while current cholesterol significantly increased in that group. For SSNS, only one case had a renal biopsy, which came back normal; however, all cases of SRNS had pathologic assessment, with 60% showing no abnormality and 24% focal membranoproliferative glomerulonephritis.

**Table 2:** Clinical, laboratory, and pathologic data of SSNS and SRNS groups

Variables		SSNS (*n* = 15)	SRNS (*n* = 25)	*P*
Clinical and therapeutic data	**Number of relapses/ years**	1 (1–2)	1 (0–3)	0.66
	**Number of hospitalization/ years**	0 (0–0)	0 (0–2)	0.01
	**Disease Duration in years**	6 (1–6)	3 (2.25–6.25)	0.43
	**Duration of steroid (months)**	36 (12–80)	39 (12–150)	0.11
	**Current dose of steroids (mg/m** ^**2**^ **/day)**	20 (15–30)	10 (0–45)	0.02*
	**Ciclosporin therapy**	0 (0%)	25 (100%)	< 0.001*
	• Duration (months)	-	27 (6–96)	-
	• Current dose (mg/kg/day)	-	3 (0–4)	-
	**MMF therapy**	0 (0%)	4 (24%)	
	• Duration (months)	**-**	23 (10–48)	-
	• Dose (mg/m^2^/dose)	**-**	400 (300–750)	-
	**Hypertension (on antihypertensives)**	0 (0%)	10 (40%)	0.006*
Vital Signs	**HR** (beat/minute)	110 (100–120)	90 (70–120)	0.002*
	**SBP (mmHg)**	110 (100–110)	110 (90–125)	0.29
	**DBP (mmHg)**	60 (50–70)	70 (50–80)	0.07
Laboratory Data	**Initial serum creatinine (mg/dl)**	0.6 (0.4–0.7)	0.5 (0.4–0.5)	0.07
	**Current serum creatinine (mg/dl)**	0.6 (0.4–0.6)	0.5 (0.5–0.6)	0.72
	**Initial serum Albumin (gm/dl)**	2 (1.8–2.8)	1.8 (1.6–1.9)	0.01*
	**Current serum Albumin (gm/dl)**	4.5 (4.1–4.7)	4 (3.7–4.25)	0.009*
	**Initial serum cholesterol (mg/dl)**	451 (420–560)	450 (355–515)	0.192
	**Current serum cholesterol (mg/dl)**	120 (110–160)	180 (151–190)	< 0.001*
	**Quantitation of proteinuria at the time of study (A/C ratio)**	0.15 (0.1–0.2)	0.1 (0–0.9)	0.62
Pathology (Renal biopsy )	• Not Done	14 (93.3%)	0 (0%)	-
	• No Abnormality	1 (6.7%)	15 (60%)	
	• Primary FSGS	-	3 (12%)	
	• Focal MPGN		6 (24%)	
	• Diffuse MPGN		1 (4%)	

**ACR**: albumin/creatinine ratio, **HR**: heart rate, **SBP**: systolic blood pressure, **DBP**: diastolic blood pressure. **focal MPGN**: focal membranoproliferative glomerulonephritis., **FSGS**: focal segmental glomerulosclerosis, **Diffuse** **MPGN**: diffuse mesangio-proliferative glomerulonephritis, **MMF**: mycophenolate mofetil, **SSNS**: steroid-sensitive nephrotic syndrome, **SRNS**: steroid-resistant nephrotic syndrome

Data are presented as median (IQR) or number (Percentage)

*: Significant *p*-value

Table 3 compares the three studied groups, control, SSNS, and SRNS, regarding M-mode and conventional pulsed wave Doppler and tissue Doppler. For M-mode parameters, LV posterior wall thickness in systole showed a significant difference between the NS and control groups (*p* = 0.014), with SRNS having the highest mean value. However, no other measurement derived from M.mode was significant, including FS and EF.

**Table 3:** Comparison between the groups regarding M-mode, conventional pulsed wave doppler, and tissue doppler

Variable		Control (*n* = 40)	SSNS (*n* = 15)	SRNS (*n* = 25)	p1	p2	Pairwise		
							**p3**	**p4**	**p5**
M.mode	**IVSd (mm)**	1.02 ± 0.32	1.07 ± 0.36	1.08 ± 0.2	0.97	0.88	0.49	0.48	0.42
	**LVIDd (mm)**	3.99 ± 0.56	4.14 ± 0.39	3.98 ± 0.5	0.60	0.95	0.40	0.62	0.42
	**LVPWd (mm)**	0.72 ± 0.09	0.75 ± 0.07	0.76 ± 0.13	0.29	0.12	0.06	0.10	0.74
	**IVSs (mm)**	1.31 ± 0.28	1.34 ± 0.26	1.38 ± 0.2	0.95	0.89	0.89	0.94	0.53
	**LVIDs (mm)**	2.49 ± 0.1	2.51 ± 0.14	2.54 ± 0.49	0.19	0.07	0.06	0.23	0.62
	**LVPWs (mm)**	1.12 ± 0.19	1.16 ± 0.16	1.25 ± 0.12	0.013*	0.014*	0.70	0.004*	0.07
	**FS (%)**	36.5 ± 8.89	39.03 ± 4.92	36.16 ± 6.65	0.55	0.72	0.32	0.86	0.28
	**EF (%)**	69.1 ± 5.9	70 ± 5.85	66.9 ± 9.8	0.90	0.83	0.56	0.91	0.87
Conventional pulsed Doppler	**MV E (cm/sec)**	81.05 ± 6.22	83.4 ± 10.8	102.4 ± 8.84	< 0.001*	< 0.001*	0.32	< 0.001*	< 0.001*
	**MV A (cm/sec)**	43.25 ± 9.03	40.6 ± 8.55	51.2 ± 8.44	< 0.001*	0.04*	0.30	< 0.001*	< 0.001*
	**MV E/A**	1.92 ± 0.29	2.1 ± 0.31	2.1 ± 0.33	0.13	0.05	0.06	0.15	0.6
Tissue Doppler	**Lateral E’ (cm/sec)**	16.3 ± 2.63	15.7 ± 1.45	16.6 ± 1.81	0.49	0.94	0.43	0.62	0.12
	**Lateral A’ (cm/sec)**	5.96 ± 1.83	6.5 ± 1.62	5.76 ± 1.9	0.22	0.67	0.17	0.71	0.07
	**E/E’**	5.13 ± 1.1	5.32 ± 0.6	6.26 ± 1.03	< 0.001*	0.001*	0.54	< 0.001*	0.003*
	**Tei index**	0.42 ± 0.04	0.43 ± 0.41	0.49 ± 0.05	< 0.001*	< 0.001*	0.56	< 0.001*	< 0.001*

**EF**: ejection fraction, **FS**: fraction shortening, **IVSd**: interventricular septum diameter in diastole, **IVSs**: interventricular septum diameter in systole, **LVIDd**: left ventricle internal diameter in diastole, LVIDs: left ventricle internal diameter in systole, **LVPWd**: left ventricle posterior wall diameter in diastole, **LVPWs**: left ventricle posterior wall diameter in systole

Data in control, SSNS, and SRNS columns are presented as mean ± SD

**p1**: Comparing the three studied groups, **p2**: Comparing Control and NS, **p3**: Comparing control and sensitive, **p4**: Comparing control and resistant, **p5**: Comparing sensitive and resistant

*: Significant *p*-value

Regarding the conventional pulsed wave Doppler, mitral valve E and A velocities demonstrated significant distinctions with an increase in NS compared to healthy children, with the SRNS group showing notably higher values for both parameters than the other groups. Nevertheless, the E/A ratio did not exhibit significant differences among groups. Concerning tissue Doppler-derived values, no significant differences were observed for lateral E’ and A’ velocities. Notably, the Tei index and E/E’ had significantly raised mean values in NS patients, prominently the SRNS, compared to the SSNS and control groups.

Color Doppler revealed trivial to mild mitral regurgitation in 2 (13.3%) cases of SSNS and 4 (16%) of SRNS, with none in controls. Therefore, the prevalence of mitral regurgitation in primary NS, although non-hemodynamically valuable, was significant in relation to controls. Aortic regurgitation was not found in any of the studied patients.

Table 4 presents a detailed comparison between the three distinct groups concerning various parameters related to 2D speckle tracking GLS and 3DE data. LV GLS was significantly decreased for NS compared to the controls in four-, three-, two-chamber, and average GLS means. Notably, the SSNS and SRNS demonstrated significantly lower GLS when compared separately with the control group, but only the four-chamber GLS and average GLS showed a significant decrease in SRNS compared to SSNS.

**Table 4:** Comparison between three studied groups regarding 2D speckle tracking derived LV GLS and 3D derived volumetric and ejection functions

Variables		Control (*n* = 40)	SSNS (*n* = 15)	SRNS (*n* = 25)			Pairwise		
					**P1**	**P2**	**p3**	**p4**	**p5**
LV strain (%)	**GLS A4C**	-22.52 ± 3.29	-20.42 ± 1.28	-18.1 ± 1.78	< 0.001*	< 0.001*	0.021*	< 0.001*	< 0.001*
	**GLS A3C**	-22.22 ± 2.43	-20.56 ± 1.44	19.69 ± 3.21	0.001	< 0.001*	0.016*	0.001	0.33
	**GLS A2C**	-21.24 ± 2.44	-19.55 ± 1.54	-18.19 ± 2.06	< 0.001*	< 0.001*	0.016*	< 0.001*	0.034
	**GLS avg**	-21.99 ± 2.55	-20.17 ± 1.29	-18.66 ± 2.16	< 0.001*	< 0.001*	0.011*	< 0.001*	0.019*
3D L V quantification	**EDV (ml)**	89.4 ± 14.6	91.6 ± 13.8	105 ± 32.6	0.064	0.079	0.39	0.024*	0.09
	**EDVI (ml/m** ^**2**^ **)**	77.8 ± 17.5	70.5 ± 26.2	92.5 ± 32.7	0.042*	0.840	0.17	0.12	0.01*
	**ESV (ml)**	53.1 ± 15.2	46.2 ± 19.8	58.5 ± 17.9	0.175	0.339	0.1	0.66	0.13
	**ESVI (ml/m** ^**2**^ **)**	22.7 ± 8.56	33.9 ± 15.9	51.9 ± 17.5	< 0.001*	< 0.001*	0.012*	< 0.001*	0.009*
	**SV (ml)**	36.4 ± 12.8	39 ± 18.4	44.8 ± 19.2	0.16	0.61	0.25	0.18	0.15
	**EF (%)**	63.69 ± 5.18	59.2 ± 6.61	56.75 ± 5.7	0.007*	< 0.001*	0.007*	0.007*	0.22

**A2C**: apical two-chamber view, **A3C**: apical three-chamber view, **A4C**: apical four-chamber view, **EDV**: end-diastolic volume, **EDVI**: end-diastolic volume indexed, **EF**: ejection fraction, **ESV**: end-systolic volume, **ESVI**: end-systolic volume indexed, **GLS**: global longitudinal strain, **SV**: stroke volume

Data in control, SSNS, and SRNS columns are presented as mean ± SD

**p1**: Comparing the three studied groups, **p2**: Comparing Control and NS, **p3**: Comparing control and sensitive, **p4**: Comparing control and resistant, **p5**: Comparing sensitive and resistant

*: Significant *p*-value

**Fig. 2:**
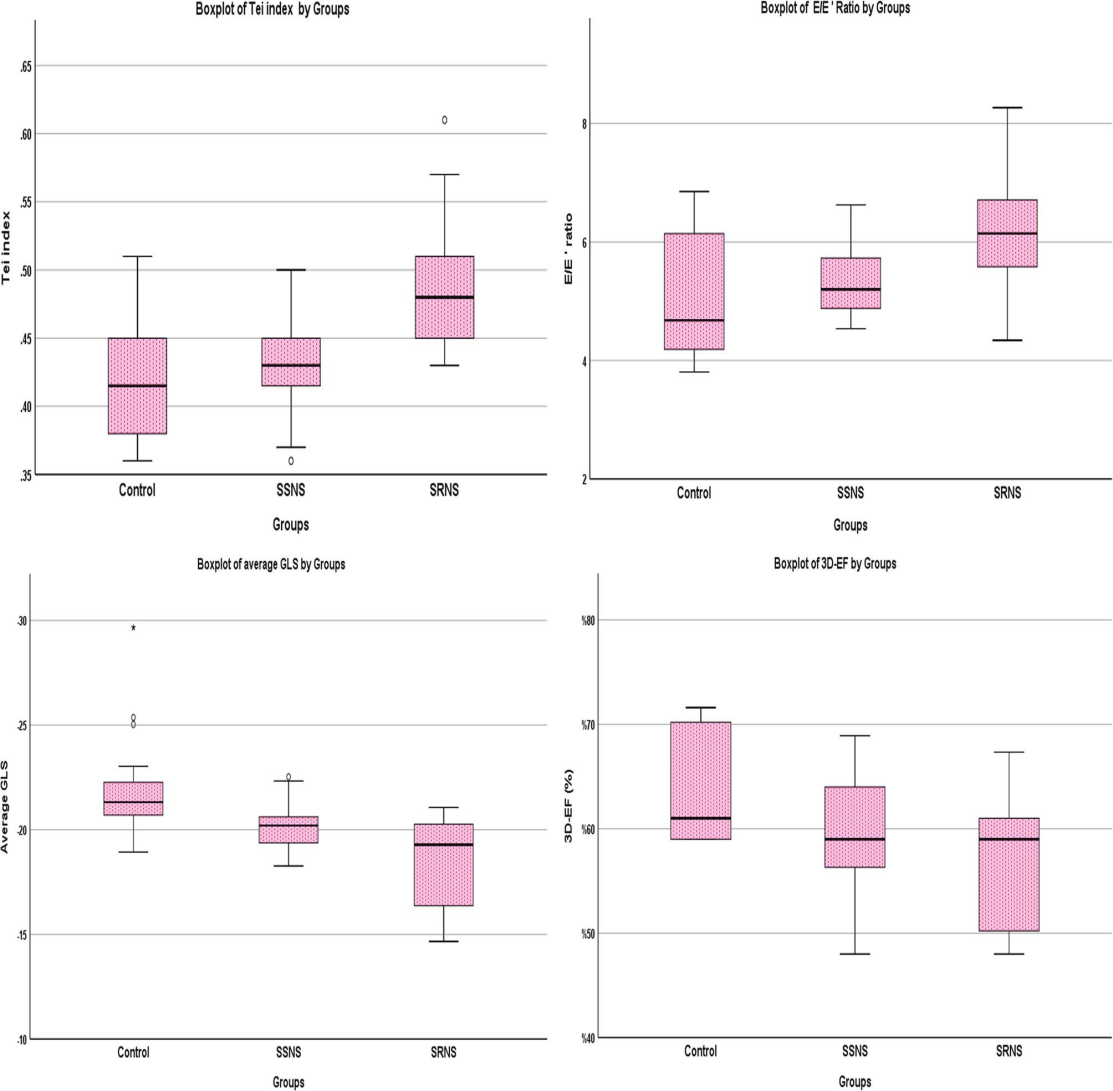
Box plot comparing the SSNS, SRNS, and controls regarding significant cardiac parameters: Tei index, E/E’, GLS, and 3D-derived EF

Concerning 3DE, it was evident that the mean 3DE-derived EF was significantly lower in NS than in healthy subjects. Moreover, a significantly low EF was detected when comparing the NS subgroups separately with controls. However, a non-significant lower EF was demonstrated for SRNS compared to SSNS. Figure 2 illustrates comparisons between the three main groups, the SSNS, SRNS, and controls, regarding some of the significant cardiac parameters: Tei index, E/E’, GLS, and 3D-derived EF.

In Table 5, Correlations are demonstrated between ECHO parameters in NS patients and disease & therapy duration, laboratory data, and relapses. Moderately positive correlations were detected between the current cholesterol level with Tei index, LV E/E’ (rho: 0.52, p: 0.001; rho: 0.59, *p*: <0.001 respectively). Also, initial serum albumin negatively correlated to a moderate extent with LV E/E’ (rho: -0.42, *p*: 0.01) and to a weak significant extent with Tei index (rho: -0.34, *p*: 0.03), while it was found to have a weak significant positive correlation with average GLS (rho: 0.32, *p*: 0.049). Another moderate strength correlation was detected between the duration of ciclosporin treatment in the SRNS group and the Tei index.

**Table 5:** Linear correlation between ECHO parameters in NS patients and disease & therapy duration, laboratory data, and relapse

	M. Mode EF (%)		LV E/A ratio		LV E/ E’ ratio		Tei index		Average GLS (%)		3DE EF (%)	
	Rho	*P*	Rho	*P*	Rho	*P*	Rho	*P*	Rho	*P*	Rho	*P*
Disease duration	0.29	0.07	0.03	0.86	-0.16	0.33	-0.15	0.35	0.034	0.84	0.095	0.56
Serum creatinine initial	0.12	0.47	-0.15	0.36	-0.17	0.29	-0.11	0.51	-0.041	0.80	-0.064	0.69
Serum creatinine current	0.16	0.33	-0.05	0.76	-0.22	0.17	-0.05	0.77	0.001	0.99	0.152	0.35
Cholesterol initial	0.18	0.28	0.10	0.53	0.16	0.34	0.17	0.29	-0.01	0.95	-0.197	0.22
Cholesterol current	0.08	0.66	0.03	0.87	0.59	< 0.001*	0.52	0.001*	-0.26	0.11	-0.033	0.84
Number of relapses (per year)	0.28	0.09	-0.01	0.96	-0.04	0.79	-0.001	0.99	-0.09	0.54	0.12	0.48
Serum albumin (initial)	-0.16	0.33	0.24	0.13	-0.42	0.01*	-0.34	0.03*	0.32	0.049*	-0.08	0.63
Serum albumin (current)	-0.26	0.11	-0.17	0.30	-0.05	0.75	-0.19	0.24	0.25	0.12	0.19	0.22
Duration of ciclosporin (months) For SRNS	0.08	0.61	-0.12	0.47	0.29	0.07	0.44	0.004*	-0.14	0.37	-0.06	0.69
Duration of steroid (months)	0.25	0.13	-0.11	0.48	-0.01	0.95	0.07	0.69	-0.13	0.43	0.012	0.94

**EF**: ejection fraction; **GLS**: global longitudinal strain, **3DE**: three-dimensional echocardiography

**rho**: Spearman’s correlation coefficient, *: Significant *p*-value less than 0.05

On multivariate linear regression analysis, significant predictors of E/E’ included current cholesterol and initial albumin levels, *p*-value = 0.019 (ß: 0.014, 95% CI: 0.003–0.26) and *p*-value = 0.003 (ß: -1.41, 95% CI: -2.28- -0.53), respectively. Nevertheless, no significant predictor among laboratory or clinical data was found upon analyzing the 3D EF, Tei index, and average GLS.

## Discussion

Patients with NS are at a heightened risk for cardiovascular disease due to frequent episodes of hypoalbuminemia, hyperlipidemia, hypertension, and the cumulative effects of steroid and immunosuppressive treatments. This study highlights the subclinical myocardial involvement in children with primary NS and the correlation with clinical and laboratory parameters. Our findings indicate significant alterations in LV functions in primary NS, particularly SRNS, compared to SSNS and healthy controls. To the best of our knowledge, this is the first study to comprehensively assess LV systolic and diastolic functions in primary NS using variable echocardiographic modalities, especially advanced techniques such as 2D-speckle tracking and 3DE with emphasis on differences between steroid response-related types of NS.

### Conventional ECHO

In the current cohort, conventional M.mode and pulsed Doppler yielded limited significant data. Only LV posterior wall diameter was thicker in primary NS, especially SRNS, compared to controls. Non-significant FS and EF that was found in the current report are consistent with other studies on NS in literature [10, 13, 18–20]. In contrast, we did not encounter significance in the E/ A ratio, while Saleh et al. and Nalcaciglu et al. found a significant decline in the E/A ratio in NS patients [18, 19].

### Tissue doppler

In our study, the LV E/E’ and Tei index were significantly higher in the primary NS cohort than in the control group, with higher means in SRNS than in other groups. Our data are consistent with a study on early NS, in which LV E/E’ was significantly higher in cases than controls despite normal conventional EF and FS [13]. Moreover, other studies have proved that the myocardial performance index (MPI) or Tei index significantly increases in NS cases [19–21].

The early manifestation of LV diastolic dysfunction may precede overt cardiomyopathy in various systemic disorders [22–24]. Tissue Doppler can configure myocardial dysfunction at earlier stages than conventional ECHO and is relatively independent of loading conditions. The combination of TDI of the mitral annulus and mitral inflow velocity from conventional Doppler (E/E′ ratio) can help distinguish the influence of preload [25–27]. E/E’ ratio is used to predict LV filling pressures; therefore, the higher E/E’ in the cases group suggests ongoing diastolic function deterioration due to impaired LV filling pressure [28], Furthermore, the tissue Doppler-derived Tei index is a collective parameter for global cardiac function evaluation, both systolic and diastolic [29], and its increase in the current cohort indicates global myocardial dysfunction.

### 2D speckle tracking

On using 2D speckle tracking, LV GLS in the primary NS cohort demonstrated statistically significant low means compared to controls. The SRNS group had significantly less GLS in both A4C and average, compared to SSNS and control groups. Our results are consistent with a study on the adult population using 2D speckle tracking with significantly low GLS in primary NS [10]. Similarly, in a study on children at early NS after 4 weeks of therapy, LV GLS derived from 3D speckle tracking was found to be significantly reduced in cases than in healthy children with hypoalbuminemia proven to be the best predictor to LV GLS and LV E/E’ [13]. Compatible findings were detected in our cohort as low initial serum albumin correlates with increased tissue Doppler Tei index and E/E’ and correlated with low GLS. On regression analysis, current cholesterol and initial serum albumin were significant predictors for E/E’ but not GLS. However, none of the two studies on adult and pediatric LV GLS differentiated subgroups according to steroid responsiveness.

The GLS is recognized as a sensitive indicator of LV systolic function deterioration in patients with various systemic diseases with apparently preserved LV EF in traditional ECHO [30–33]. Lower GLS values are also linked with higher mortality risks in the general population [34].

### 3D echocardiography

Despite the non-significant differences in conventional EF between NS and controls, 3D ECHO-derived EF demonstrated a whole different story. Systolic dysfunction was evident, and the NS group had a statistically significant low EF. The 3D volumetric evaluation of LV function is far more accurate than traditionally measured EF, with comparable results to EF and volumetric evaluations measured via cardiac magnetic resonance [35]. Therefore, based on the precedent finding, we could argue the opinion of some researchers that in primary NS, systolic functions are preserved [18]. No previous research in the literature addresses LV 3D EF in pediatric NS cohorts.

### Suggested etiologies for LV impairments

Although we detected numerous functional impairments in LV of NS children, no single etiologic factor could be incriminated. Beside the known effects of steroids and immunosuppressives, in an induced NS rat model, researchers found cardiac muscle atrophy and reduced LV contractility after two weeks of NS. This cardiac atrophy was associated with increased cell size but showed no significant myocardial fibrosis. Elevated myocardial expression of aquaporins contributed to cardiomyocyte edema, causing myocardial stiffness and contractile dysfunction. Additionally, inflammatory cytokines like TNF-α and IL-1β were elevated, with increased phospholamban, a protein involved in calcium regulation, contributing to cardiac remodeling and dysfunction [36, 37]. The significant levels of TNF are a well-known implication of NS [38, 39]. It was used to target its receptor in NS in some of the therapeutic protocols [40]. Moreover, TNF has been proven to have a negative inotropic effect and prolonged activation of TNF-alpha and its soluble receptors in heart failure patients, whether compensated or decompensated [41–43]. Decreased albumin level was suggested to induce myocardial dysfunction as albumin was found to have a role in scavenging free oxygen radicals and decreasing inflammation, while decreased albumin has a pathogenic impact in developing atherosclerosis and endothelial dysfunction, which might impair coronary perfusion and subsequently impair the ventricular function [44–46]. Another suggested factor is possible increasing evidence of accelerated atherosclerosis in children with persistently high lipid levels [47]. The albumin and cholesterol levels in the current cohort were correlated with several cardiac functional parameters.

### Study limitations

This study has several limitations. The single-center design with a relatively small sample size warrants a multicenter design with variable patient demographics, socioeconomic backgrounds, and therapeutic differences. Furthermore, the lack of blinding may have introduced bias in evaluating the outcomes. In future research, blinding in outcome assessment is recommended to reduce evaluation bias. Moreover, the study duration was relatively short, with each patient being assessed using ECHO only once. Larger studies with extended long-term follow-up periods are necessary in the future to understand better the early cardiac dysfunctional changes and their impact on the overall prognosis of children with NS. Additionally, long-term studies would assess the lasting effects of NS on LV functions. Given the multifactorial nature of cardiac dysfunction, it is possible that not all confounding variables were adequately controlled, which may have influenced the findings. Therefore, it is recommended that more confounding variables be stringently controlled in upcoming research to improve the accuracy of research results.

## Conclusion

Subtle subclinical left ventricular myocardial dysfunctional alterations, either diastolic or systolic, occur in pediatric patients with primary NS and are detectable using variable echocardiographic modalities, including 3D-derived echocardiographic parameters with substantial differences upon comparing SSNS and SRNS. Furthermore, these parameters significantly correlate and could be predicted via disease-related biochemical changes. Therefore, it is reasonable to consider including advanced cardiac functional screening in primary NS cases.

## Data Availability

Not applicable.

## Data Availability

The datasets used and/or analyzed during the current study are available from the corresponding author upon reasonable request.

## Abbreviations

BMI: Body mass index
CNI: Calcineurin inhibitors
ECHO: Echocardiography
EF: Ejection fraction
GLS: Global longitudinal strain
LV: Left ventricle
MMF: Mycophenolate mofetil
SV: Stoke volume
NS: Nephrotic syndrome
SRNS: Steroid-resistant nephrotic syndrome
SSNS: Steroid-sensitive nephrotic syndrome
2D: Two-dimensional
3DE: Three-dimensional echocardiography
